# Soil Fertility Improvement with Mixtures of Wood Ash and Biogas Digestates Enhances Leaf Photosynthesis and Extends the Growth Period for Deciduous Trees

**DOI:** 10.3390/plants12051152

**Published:** 2023-03-03

**Authors:** Austra Zuševica, Aleksandrs Adamovičs, Kārlis Dūmiņš, Viktorija Vendiņa, Sindija Žīgure, Dagnija Lazdina

**Affiliations:** 1 Latvian State Forest Research Institute SILAVA, 111 Riga St., LV-2169 Salaspils, Latvia; 2 Faculty of Agriculture, Latvia University of Life Sciences and Technologies, 2 Liela St., LV-3001 Jelgava, Latvia

**Keywords:** by-products, poplar, fertiliser, CCI, SLA, photosynthetic rate, digestate, ash

## Abstract

In the context of climate change, it is necessary to establish forest management by balancing more products, using less area, and minimizing environmental impacts. The use of different industrial bio-based by-products as soil conditioners in the last few decades has gain more interest, because it leads to an extended use time of these products and supports the circular economy. The aim of this study was to determine the effect of fertiliser made from cattle and pig manure biogas fermentation digestate and wood ash from two cogeneration plants, applied in different mixture ratios, to test its suitability for fertilisation of deciduous trees, using the physiological, morphological, and chemical parameters of the leaves as an indicator. We selected two poplar clones: foreign ‘OP42’ (syn. Hybrid 275) and local ‘AUCE’ annual shoot stem cuttings as planting materials. A negative control group with acidic forest mineral soil as substrate and four fertilised groups with different applied digestate and wood ash ratio mixtures to forest soil was established (ash:digestate 0:0 (Control), 1:1, 2:1, 3:1, 4:1). Mixture application improved growing conditions because all fertilised group poplars had longer growth periods and photosynthetic rates in August than the control group. Both local and foreign clones showed a good response to fertilisation in terms of leaf parameters. Poplar is a suitable culture to fertilise with bio-waste biogenic products, because of its capacity to absorb nutrients and fast response to fertilisation.

## 1. Introduction

Increasing global waste products requires more solutions and technologies for their sustainable management, including opportunities for more efficient disposal, but also for their reuse. The utilization of solid organic waste (SOW) substances is governed by regulatory frameworks. SOW has a great potential for its reuse, for example, in soil fertilisation and energy production; therefore, in recent years SOW is globally viewed as a resource and not a waste [1,2,3]. The use of waste materials as soil conditioners is an example of a circular economy, because it returns the product into the economy once again, as well as compared with mineral fertilisers it can be more cost-effective and helps to minimize the environmental impact associated with mineral fertilisation [4,5]. Usually, SOW has high nitrogen (N), phosphorus (P), and potassium (K) contents, and are therefore suitable for improving the soils to gain higher primary production as well as provide better microbial activity [6].

Biogas production is a viable renewable energy source, and it also provides multiple environmental benefits [7]. In Europe, biogas plants have developed as a solution for sustainable waste disposal and a way to become more energy-independent [8]. Due to the growing consumption and intensification of agriculture in Europe, both the amount of substrate used and the number of biogas plants themselves are expected to increase [9,10]. In biogas plants, digestate is formed as a waste product in the production process [11]. The digestate makes up almost the same mass as the primary substrate used in the digestion process, and this waste product is legally classified as hazardous in some countries and European regulation states that it needs to be disposed of. During biogas production, two fractions of digestate are formed—liquid and solid. Both of these fractions have a high NPK content and have the potential to be used as fertiliser [11,12], although there are studies debating whether solid digestate contains the appropriate amount of N and should be used as fertiliser and N content in the liquid digestate fraction is higher [13]. The use of the solid fraction of digestate improved soil physicochemical properties and provided longer and less concentrated plant absorption of nutrients due to the higher content of lignin in the solid fraction [7,14,15]. This is particularly important if this product is intended to be used in natural or semi-natural ecosystems, thus minimizing the risk of nutrient leaching and environmental eutrophication.

A wide variety of biological materials can be used as substrates in biogas production: animal manure, algae, municipal solid waste, energy crops, food waste, sewage sludge, and animal by-products [16,17,18,19]. The price of biogas production as well as the chemical composition of digestate differs depending on the origin of the substrate [15,20,21]. In Europe, agricultural waste, including manure and food waste, are commonly used as substrates for biogas production [22]. It has been previously shown that pig or cow manure substrate used in biogas production did not affect the chemical composition or microbiological activity of the digestate, but there is a lack of information on whether it is likely to affect the nutrient uptake capacity of plants, and thereby the physiological and morphological parameters of the leaves [23].

The cogeneration plant on woodchips also forms a by-product—wood ash—which can be used as a fertiliser and liming agent. Ash chemical composition varies depending on the substrate as well as the burning temperature [24]. Fertilisation and liming of acidic soils improve the soil mineral content and their availability to plants, resulting in improved plant growth and vitality parameters [25]. The use of wood ash as a soil conditioner improved planted tree survival rates and growth parameters in organic soils with high N, but low P and K content [26]. However, fertilisers containing high N content are required to improve growing conditions in poor mineral soil with low NPK levels. The number of studies about the use of these two by-products as a plant fertiliser has increased in recent decades. However, although the use of wood ash as a fertiliser and liming agent in tree plantations has been studied, studies of the use of biogas digestate as plant fertiliser mainly focus on crops, and there are few of studies about the effect of soil conditioning with digestates on tree growth [27,28,29,30,31]. In addition, fertilisation with digestates has already shown a positive effect on the growth of poplars and willows, which is a fast-growing tree species [32].

Adjusting bio-based by-product use for tree fertilisers supports both the problems with by-product utilization along with finding a new alternative to mineral fertilisers for soils with low nutrients in natural and semi-natural ecosystems. Therefore, this practice creates a circular bioeconomy system. This study aimed to determine the effect of the application of a new fertiliser mixture with different ratios of a biogas fermentation and wood cogeneration plant residue, to improve growth conditions for foreign and local poplar clones on low-nutrient acidic soil. The study used a solid fraction of digestate derived from the local biogas plants, in which pig and cattle manure was used as a primary substrate in the biogas production. Wood ash was derived from wood chip generation plants. The physiological parameters of the leaves fit a reliable assessment to observe the current effect of external factors on tree growth and vitality. This study addresses the following research questions: (1) Does the effect of biogenic fertilisation of foreign and local poplar clones differ; (2) Does the origin of the ash and primary substrate of the digestate affect the physiological parameters of the leaves; (3) Which selected biogas digestate:wood ash ratio is more effective for tree fertilisation in the context of leaf phenological and morphological parameters. The chemical, morphological, and physiological parameters of the leaves were measured to answer the questions raised in the study. The C, N, and P contents of the leaves was determined; the fresh and dry biomasses were measured; the Specific Leaf Area (SLA) index was calculated; and the chlorophyll content was measured three times during the growth period and the photosynthetic rate was measured twice during the growth period with non-invasive methods.

## 2. Materials and Methods

### 2.1. Experimental Design

**Figure 1:**
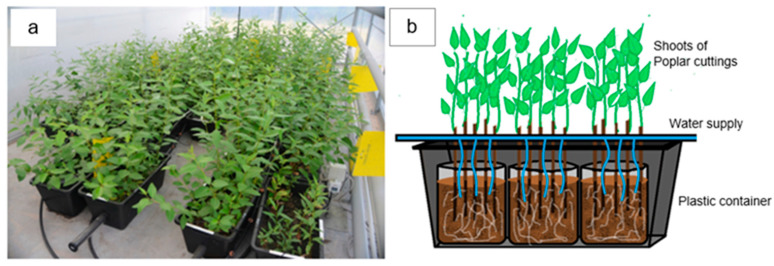
(**a**) Poplar cuttings planted in semi-controlled conditions in plastic bags which were put into plastic containers in the greenhouse. (**b**) Illustration of a plastic container that contains three soil bags wherein five poplar cuttings are planted in each and are supplied with water by an automatic watering system.

To test the efficiency of wood ash and digestate mixtures for the fertilisation of two poplar clones, an experiment was carried out in a semi-controlled greenhouse in the Latvian State Forestry Research Institute “Silava”, Latvia, (N 56°870081′′ E 24°347465′′). The experiment was set up in mid-April but finished in mid-September. Shading and daylight length were not controlled and was the same as the natural conditions in this region. Plants were supplied with water using an automatic watering system which applied water to all plants equally, depending on weather conditions (Figure 1a,b). The temperature varied depending on the month and weather conditions outside. The mean temperature in the nearest meteorology station (27 km from the experimental greenhouse) was 6.0 °C in April, 11.1 °C in May, 20.0 °C in June, 22.2 °C in July, 16.1 °C in August, and 10.8 °C in September.

Two poplar clones were selected for the experiment: the widely used clone ‘OP42’ (syn. Hybrid 275) and the clone ‘AUCE’ selected in Latvia. For vegetative propagation, 20 cm long and 1.0–1.5 cm in diameter stem cuttings were prepared. Cuttings were directly planted in 20 L volume and 30 cm depth soil bags with holes at the base (Figure 1a,b). Five stem cuttings were planted per one plastic bag. Three soil bags were put in one plastic container. Plastic containers were a closed system, to prevent the leaching of fertiliser. The substrate was acidic (pH = 4.3), nutrient-poor, forest mineral podzol soil with low K and P values collected from a meliorated young conifer stand (Table A1). It was supplemented with mixtures with different proportions of biogas digestate and wood ash: the mixture of digestate (D) (cattle manure digestate from JSC “Ziedi JP” and pig manure digestate from LLC “Gren Jelgava”) and wood ash (P) (from LLC “Gren Latvia” (a) and LLC “Dobeles Eko” (b)) at different proportions (P+D 1:1; P+D 2:1; P+D 3:1; P+D 4:1). In addition, a control group in which the poplar cuttings were planted in unfertilised forest soil was established (Table 1 and Table A2).

### 2.2. Data Collection

**Figure 2:**
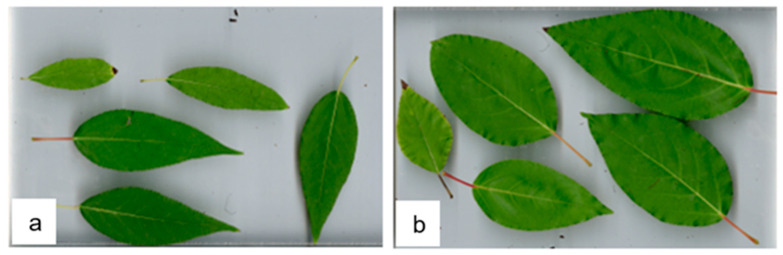
Morphological differences between leaves of different poplar clones (**a**) ‘AUCE’, (**b**) ‘OP42’.

The Chlorophyll Content Index (CCI) of the leaves was measured by a non-invasive method using a near-infrared ‘The SpectraVue’ leaf spectrometer (CID Bio-Science, Camas, WA, USA). CCI measurements were performed three times during the growth period on the 8th of June, the 8th of July, and the 20th of August. The photosynthetic rate of the leaves indicates the performance of both photosystems—Photosystem I and Photosystem II. It indirectly shows the plant’s capacity for carbon fixation. The photosynthetic rate was also determined by a non-invasive method using an LC-proSD (ADC Bioscientific Limited, Hoddesdon, Hertfordshire, UK) mobile infrared gas analysis photosynthesis system. Measurements were taken twice during the growth period—at the beginning of the growth period from the 6th to the 10th of June, and at the end of the growth period from the 24th to the 27th of August. The measurements were performed from morning (7.00 a.m.) until noon (11.30 a.m.). For photosynthetic rate and CCI measurements, three shoots from each group were chosen. From each shoot, three healthy sun leaves of similar size from the top part of the shoot were selected for photosynthetic rate and CCI measurements. The first five younger leaves at the top of the shoot were not selected for measurements, because photosynthesis processes in juvenile leaves can vary significantly from mature leaves. The analytical chamber environment was established at 25 °C and with an LED mixed red and blue light unit to PAR of 900 m^−2^ s^−1^ which is similar to the light amount on a sunny day with clouds. The mean external CO_2_ concentration was 378 ppm in June and 452 ppm in August. Each leaf was acclimatized in the analytical chamber at least for 5 min before the measurement was performed. Shortly after the final measurements of chlorophyll content and photosynthetic rate, all leaves from the measured shoots were selected. Five leaves of each sampling tree, from the first fully developed leaf from the top of the shoot and in a downward direction, were scanned with the ‘Winfolia2019’ software program (Regent Instruments Inc., Québec City, QC, Canada) (Figure 2a,b). The total photosynthetic capacity of a tree was calculated by multiplying the mean leaf photosynthetic rate and mean leaf area. After the determination of fresh mass, the sample was dried at 40 °C for 10 days. Using the leaf area data obtained from the scanned leaves and leaf dry mass, the Specific Leaf Area (SLA) was calculated (the ratio of leaf area and dry mass). 

The chemical compositions of substrates and leaves were analysed in the Latvian State Forest Research Institute ′′Silava′′ Forest Environment Laboratory (Accreditation No. LATAK-T-631-02-2020). Newly made wood ash and biogas digestate mixtures were analysed before applying to the soil on the 29th of April (Table A1). Soil analyses were carried during the final physiological data measurements at the end of the growth period, from 26th to 30th of August (Table A2 and Table A3). For each group (clone, the primary substrate of digestate, ash origin, digestate and ash ratioand ‘BMT Ecocell 55’ laboratory dryer (BMT Medical Technology s.r.o., Brno-Zábrdovice, Czech Republic) (Normative-technical documentation No. LVS ISO 11465:2002). Total C and N contents in substrates and leaves were determined using an ‘Elementar El Cube’ elemental analyser (Elementar, Langenselbold, Germany), and total P content was measured using a ‘Shimadzu UV-1900’ spectrometer (Shimadzu, Kyoto, Japan) (Normative-technical documentation No. LVS ISO 10694:2006 L; LVS ISO 13878:1998; LVS 398:2002 L). For substrate pH elevation a ‘Adrona AM 1605’ pH meter (Adrona, Riga, Latvia) was used (ISO 10390:2021). For the combined K, Ca, and Mg analyses, a ‘Thermo Fisher Scientific iCAP 7200 Duo’ Inductively Coupled Plasma Optical Emission Spectroscopy (ICP-OES) spectrometer (Thermo Fisher Scientific, Waltham, MA, USA) was used (Normative-technical documentation No. LVS EN ISO 11885:2009).

### 2.3. Data Analyses

The computer software R Statistics 4.0.5. (Gentleman R. and Ihaka R., Vienna, Austria) was used for statistical analyses and data visualization [33]. Morphological data such as leaf area, SLA, and leaf chemical parameters were tested with the ‘Shapiro’ test and did significantly differ from a normal distribution, and therefore non-parametric statistical analyses were chosen. Additionally, leaf physiological data differed significantly from a normal distribution. Therefore, for all leaf physiological data, non-parametric statistical analyses were chosen. To test significant differences between two factors, the ‘Kruskal–Wallis’ test was used, but to determine differences between groups the ‘Pairwise Wilcox’ test was performed. Correlation analyses and correlation matrices were made using the R library packages ‘Hmisc’ and ‘corrplot’ [34,35]. The correlation was made using the Pearson correlation coefficient.

## 3. Results

**Figure 3:**
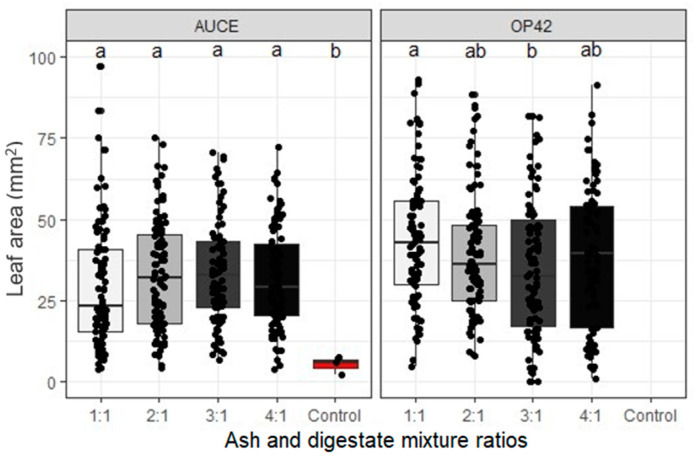
Influence of different ratios of wood ash and digestate on leaf area (mm^2^) for ‘AUCE’ and ‘OP42’ poplar clones. Different letters indicate significant differences (*p* < 0.05) using the ‘Pairwise Wilcox’ test.

Poplar clone ‘OP42’ leaves have a different form and larger mean leaf area than the ‘AUCE’ clone (*p*-value < 0.001), and were therefore analysed separately (Figure 2a,b). ‘AUCE’ clone leaf size was not significantly affected by the primary substrate of biogas digestate, but it was affected by the ratio of the mixture (*p*-value = 0.0083) (Figure 3). All leaves in the fertilised groups had larger areas, but there were no differences between the different ratios of the mixture (1:1 vs. Control *p*-value = 0.017, 2:1 vs. Control *p*-value = 0.016, 3:1 vs. Control *p*-value = 0.016, 4:1 vs. Control *p*-value = 0.016). Additionally, ‘OP42’ group leaves were not affected by the primary substrate of digestate, but there was an effect depending on the ratio of the mixture (*p*-value = 0.0337) (Figure 3). The average leaf area of the mixture ratio 1:1 was larger than for 4:1 (*p*-value = 0.024).

**Figure 4:**
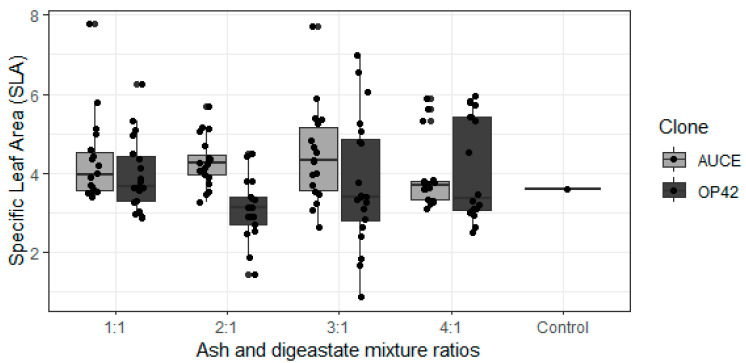
Specific Leaf Area (SLA) depending on ratio of the wood ash and digestate and poplar clone.

SLA was not influenced by the fertiliser mixture ratio, the primary substrate of digestate, or the origin of the wood ash. SLA was higher for the ‘AUCE’ clone than for ‘OP42’ (*p*-value = 0.0006) (Figure 4). The ‘AUCE’ clone control group had a lower mean value than the fertilised groups, but leaves from the ‘OP42’ clone control group were not measured, as they had already fallen off at the time of data collection.

**Figure 5:**
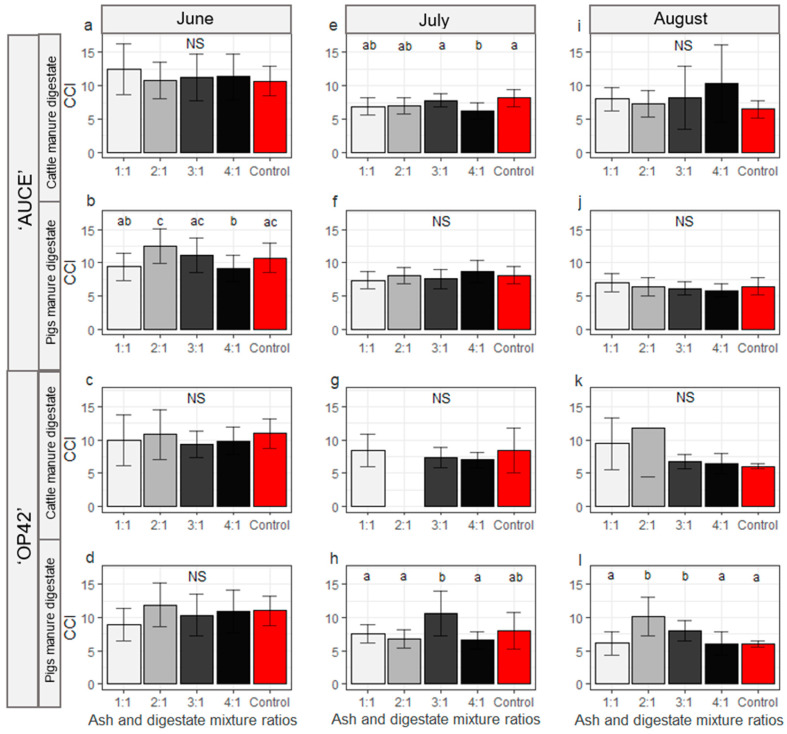
CCI (Chlorophyll Content Index) depending on the different ratios of the wood ash and digestate mixtures in June (**a**–**d**), July (**e**–**h**), and August (**i**–**l**) for ‘AUCE’ (**a**,**b**,**e**,**f**,**i**,**j**) and ‘OP42’ (**c**,**d**,**g**,**h**,**k**,**l**) clones fertilised with digestate whose primary substrate was cattle (**a**,**e**,**i**,**c**,**g**,**k**,) or pig (**b**,**f**,**j**,**d**,**h**,**l**) manure. Letters represent significant differences (*p* < 0.05, pairwise Wilcox test). The population standard deviation is represented with error bars.

The primary substrate of digestate did not affect the CCI (*p*-value = 0.08742). The CCI was significantly higher in the ‘OP42’ clone; therefore, these clones were further analysed separately (*p*-value = 0.04566). The CCI was influenced by the month of the growth period (*p*-value < 0.0001). The highest CCI was in June and it decreased towards the end of the growth period (June–July *p*-value = 0.0001, July–August *p* = 0.0092). In June, the CCI was influenced by the poplar clone (higher for the ‘AUCE’ clone than ‘OP42’ *p*-value = 0.03877), but the primary substrate of digestate did not affect it. Chlorophyll content differed between mixture ratios in ‘AUCE’ clone poplars fertilised with pig manure digestate (*p*-value = 0.0006), and a higher chlorophyll content index was obtained from the 2:1 and 3:1 mixture ratios (Figure 5b). For the ‘OP42’ clone there were no significant differences between the primary substrate of digestate and ratios of the mixture during June (Figure 5c,d). In July, the CCI was not influenced by the poplar clone. For ‘AUCE’ clones fertilised with cattle manure digestate, 4:1 had significantly lower CCI content (*p*-value = 0.0043, 4:1 vs. 3:1 *p*-value = 0.0098, 4:1 vs. Control *p*-value = 0.0256) (Figure 5e). For the ‘OP42’ clone, the ratios of the mixture from cattle manure digestate did not affect CCI, but poplars fertilised with pig substrate digestate had the highest CCI content in the 3:1 mixture ratio (*p*-value = 0.0046; 3:1 vs. 1:1 *p*= 0.0354; 2:1 *p*-value = 0.0354, Control *p*-value = 0.0038) (Figure 5g,h). In August, the ratio of the mixture affected CCI only for the ‘OP42’ clone fertilised with pig manure digestate, and it was lower in the 1:1, 4:1, and control groups (*p*-value = 0.0003982, 1:1 vs. 2:1 *p*-value = 0.0044, 1:1 vs. 3:1 *p*-value = 0.0189, 2:1 vs. 4:1 *p*-value = 0.0061, 2:1 vs. Control *p*-value = 0.0044, 3:1 vs. Control *p*-value = 0.0044, 4:1 vs. Control *p*-value = 0.05) (Figure 5l).

**Figure 6:**
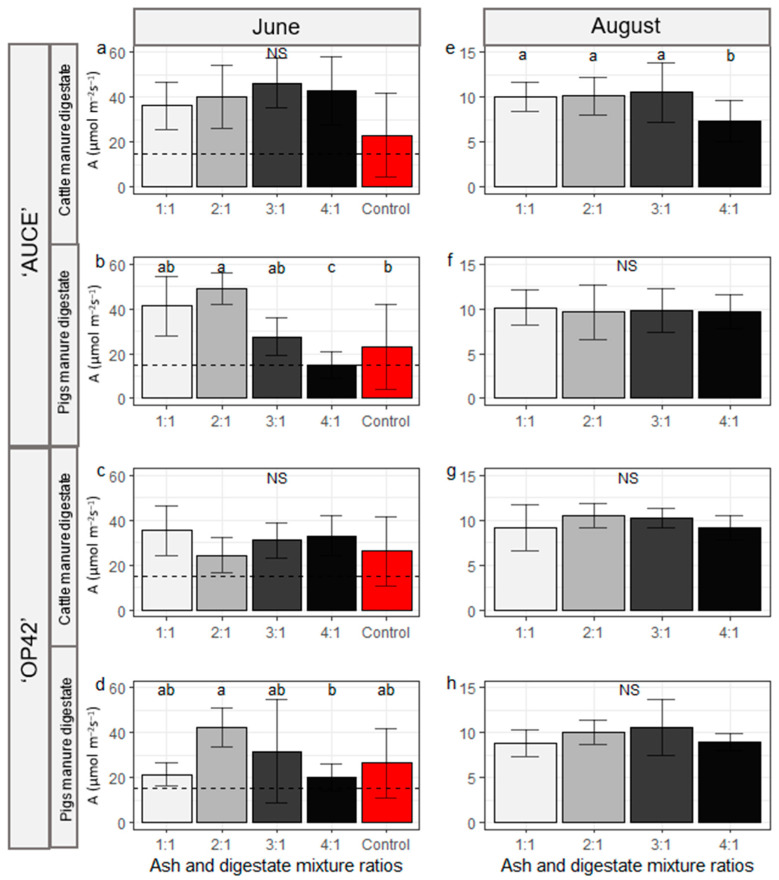
A (Leaf photosynthetic rate) in June (**a**–**d**) and August (**e**–**h**) for poplar clones ‘AUCE’ (**a**,**b**,**e**,**f**) and ‘OP42’ (**c**,**d**,**g**,**h**) fertilised with cattle (**a**,**e**,**c**,**g**) or pig (**b**,**f**,**d**,**h**) manure digestate depending on the different ratio of the wood ash and digestate mixtures. For June and August plots, different *y*-axis length was chosen to better represent differences in both months. The maximum of the *y*-axis scale differs between months and the black dashed line in June plots represents y axis length in August plots. Letters represent significant differences (*p* < 0.05, pairwise Wilcox test). The population standard deviation is represented with error bars.

In June, there were no significant differences in leaf photosynthetic rate for both poplar clones fertilised with cattle manure digestate, but for poplars fertilised with pig digestate the control group and 4:1 had the lowest rate, but the 2:1 mixture ratio had the highest rate of photosynthesis (‘AUCE’ clone: *p*-value < 0.0001, 4:1 vs. 1:1 *p*-value = 0.0008, 4:1 vs. 2:1 *p*-value = 0.0008, 4:1 vs. 3:1 *p*-value = 0.0052, 3:1 vs. Control *p*-value = 0.0297; ‘OP42’ clone: *p*-value = 0.1227, 4:1 vs. 2:1 *p*-value = 0.008) (Figure 6a–d). In August, photosynthetic rate was measured only in the fertilised groups, because all control group leaves had either already fallen or the leaf area was too small for measurement. Significant differences between the ratios of the mixture were observed only for the ‘AUCE’ clone fertilised with cattle manure digestate, where the photosynthetic rate was lower in the 4:1 ratio group compared to the other groups (Figure 6e–h).

**Figure 7:**
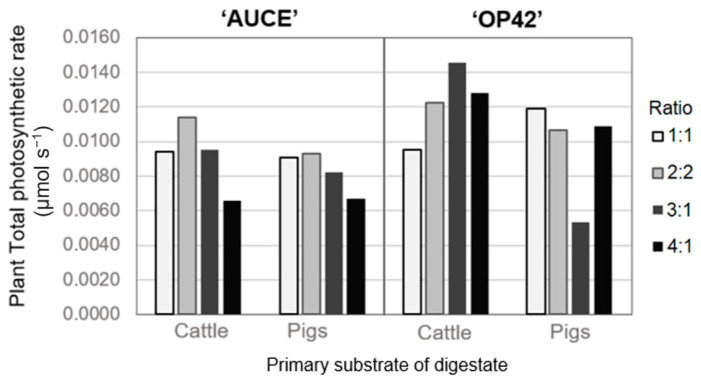
Leaf total photosynthetic rate in August depending the clone of poplar, primary substrate of digestate, and different ratios of wood ash and digestate.

The total photosynthetic rate was calculated only for the fertilised groups because there was no data on the control group’s photosynthetic rate in August. For the ‘AUCE’ clone plants, the total photosynthetic rate was similar for both primary substrates of digestate (cattle manure and pig manure), but it differed for the ‘OP42’ clone in the 3:1 mixture ratio (Figure 7). Overall, the mixture ratio with the smallest amount of wood ash (one or two parts wood ash and one part biogas digestate) had the most stable increase in photosynthetic rate, and therefore were the ratios with the most potential for use in fertilisers.

**Figure 8:**
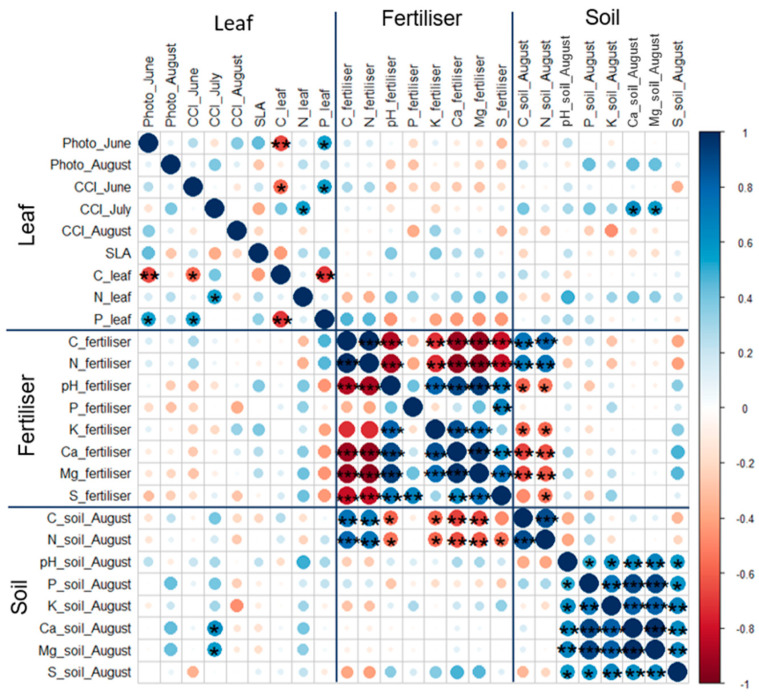
Correlation matrix, made using ‘corrplot’, between leaf physiological and morphological parameters and fertiliser and fertilised soil chemical composition in August (abbreviations: Photo—Photosynthetic rate (μmol m^−2^ s−^1^), CCI—Chlorophyll Content Index). Significance level: * *p* = 0.05–0.01, ** *p* = 0.01–0.001, *** *p* < 0.001.

Leaf photosynthetic rate and CCI in June had a positive correlation with leaf P content (*R* = 0.55, *p* = 0.028; *R* = 0.56, *p* = 0.024), but they did not correlate with leaf N content (*R* = 16, *p* = 0.56, *R* = 0.096, *p* = 0.72) (Figure 8). However, both of these leaf physiological parameters negatively correlated with carbon content in leaves (*R* = 0.66, *p* = 0.0056; *R* = 0.57, *p* = 0.021). The photosynthetic rate was lower and chlorophyll degradation started in August, and both of these parameters did not correlate strongly with other leaf parameters (SLA and leaf C, N, P contents). However, leaf photosynthetic rate had a positive correlation (*R* < 0.54) with soil P, Ca, and Mg contents in August.

## 4. Discussion

### 4.1. Choosing between Foreign and Local Poplar Clones

The clones investigated in the study had differences in plant strategies for nutrient allocation in different plant parts, and also in terms of responses to survive and compete in certain climatic conditions. In the hemi-boreal zone, when choosing the poplar hybrid with the best genetics for tree stands, there is usually a need to decide on a trade-off between better resistance against frost damage and a better growth rate [36]. Usually, poplar hybrids selected from native species show better survival rates during extremely cold winters in the region, while clones and variations with origins in southern regions have faster growth rates and better productivity [37,38]. Nonetheless, we will face more climatic complications due to climate change in the near future and also presently; therefore, the critical factors by which we choose the best variation for current tree stand establishment should also include the resistance from existing and future biotic and abiotic threats in the region [39,40]. Due to the intensification of local climate extremes, the local hybrids or hybrids that are adapted to the local climate should have better survival and acclimatization capacities due to the importance of phenology [41]. Therefore, the weight of these trade-off factors should consider whether it pays off to focus on productivity, or in the changing climate, whether the productivity differences will be negligible in the context of the increasing threats to tree survival [42]. It is important to see the differences in productivity between foreign and local clones growing in the same climatic conditions to increase our knowledge and create more effective fact-based decision making for the selection of plant species in the future. 

SLA is one of the most widely used plant leaf traits and it shows how much leaf photosynthetic area is present on each leaf biomass unit [43]. Foreign clone ‘OP42’ had a lower SLA. Therefore, SLA decreases in leaves with a thicker cell wall and with more stored non-structural carbohydrates [44,45], a lower SLA value indicates that more biomass investments are made for foliar support per leaf area. In this study, this was also supported by the data on the C content in leaves, which was higher in the ‘OP42’ leaves (Table A3). Plants with more dry mass and carbon stored in the leaves have a lower photosynthetic rate because the carbon content in the leaf negatively correlates with photosynthetic rate and CCI. Plant traits like lower SLA and higher N/P, which was present in ‘OP42’, are usually related to higher latitudes [46]. Overall, the OP42 clone showed higher leaf area, CCI, and plant total photosynthetic rate in August, which indicates higher productivity than the local ‘AUCE’ clone. However, it is known that ‘OP42’ does not have high resistance to frost damage in the cold winters in this region, and is therefore only suitable for planting in areas where winter temperatures do not drop very low [38].

### 4.2. The Potential of Digestate and Wood Ash Mixtures

Wood ash origin (from two wood chip cogeneration plants) and the primary source of biogas digestate (cattle or pig manure) did not affect most morphological and physiological parameters. This indicates that the variation of nutrient composition in the mixture does not significantly vary from the origin of the primary substrate and that the result can be expanded into a wider context, with similar results already published in other studies [23]. Leaf physiological parameters, CCI (which correlates with leaf chlorophyll content), and photosynthetic rate varied significantly depending on the time in the growth period and the highest was in June when then decreased towards the end of the growth period [47].

Although the mixture ratios of ash and digestate did not significantly affect the SLA, it was lower for the ‘AUCE’ clone in the control group and the group with the highest ash content. SLA tends to be dependent on nutrient availability in the soil and is lower in plants grown in nutrient-poor soils [48]. The soil and leaf chemical analysis results showed lower soil and leaf N and P contents in the group with the highest ash content, but stored leaf carbon, although variable, showed a higher amount in this group (Table A2 and Table A3). Chemical analyses of the control group could not be performed due to the small number of leaves that survived until August. However, because of the similar SLA results, it can be speculated that the decreased SLA parameter in both of these groups (Control, 4:1) was influenced by low N and P contents in the soil, which led to the formation of leaves with less photosynthetic area per biomass unit [44]. Although liming increases soil pH values, which promotes better accumulation of minerals in the plant, it also activates N mineralisation [49]. Soil analyses at the end of the growing season showed that the nitrogen value in 4:1 was lower than in other groups, which could negatively impact the growth of nitrophilous species such as poplars (Table A2) [50]. The CCI content was very variable throughout the growing season, with one of the lowest values in the group with the highest wood ash content and the control group; the same results were observed in photosynthetic rate during June and August [51]. This all indicates that fertilisation with a mixture increases the photosynthetic rate, but a large ash proportion does the opposite. Moreover, in June, it was possible to measure the photosynthetic rate of the leaves, but due to the leaves already falling off in the control group at the beginning of August, it was not possible to take these measurements for the control group. However, the fact that the leaf fall had begun much earlier for the unfertilised control group indicates the prolonged growth period in the autumn for the fertilised groups [52]. Additionally, the total plant photosynthetic rate, which was calculated by using the leaf area, number of leaves, and photosynthetic rate in August, showed that the group with the best results for both clones and primary substrates of digestate was the two ash and one digestate proportion, but groups with three and four proportion of ash sometimes showed a rapid decrease [53]. From the results of this study, it could be suggested that the two mixture ratios with the smallest wood ash proportion should be used for increased productivity without negatively impacting the environment, because this method not only significantly increases the physiological vitality of the plant, but also reduces the threat to biogenic pollution in the ecosystem.

These results indicate that the waste products can be valorised and reintroduced back into the economy, and therefore entering the scope of a circular economy [54]. Climate change predicts new solutions to enduring a more climate-neutral Europe. It is important not only to find and established new and innovative energy production technologies that are not suitable for all countries due to economical and geographical differences, but also fully utilise local resources and adopt the most appropriate energy production system for each region. [55]. Biomass production is one of the main local energy resources in northern countries without meaningful mineral resources [56]. By using modern technologies, it is possible to manage biomass energy in a way that is good for the environment, and one of these aspects is reusing waste products [56]. Evolving the use of local energy production resources helps to increase states’ energetic independence [55]. Poplar is a promising wood production species to grow by fertilising with biogenic fertilisation due to its phytoremediation capacity because poplars can store most of the nutrients from the soil in plant parts, and therefore mitigate the threat to ecosystem eutrophication and nutrient leaching [57]. However, this study indicates the advantages of using bio-waste mixtures to improve leaf physiological parameters, which indicates increased productivity. The economic benefits of wood chip cogeneration and biogas production depend more heavily on factors that change over time, such as electricity prices, the consumption of generated thermal energy, as well as on political initiatives, but can also be influenced by the usage of digestates as soil fertilisers [58]. To determine the economic advantage of the application of this method, a comprehensive study including the cost of logistics, production of fertiliser, and mechanical application would be needed.

## 5. Conclusions

Foreign clone ‘OP42’ had a higher productivity as shown by leaf parameters such as larger leaf areas, and higher CCI and photosynthetic rate, and hence, it could be used as an alternative to local clones. The low resistance of ‘OP42’ to frost damage at the juvenile age described in earlier studies, should be carefully considered in decision making. The earlier end of the growth period and small leaf area of the control group poplar leaves indicates the relevant impact of fertilisation on poplar cuttings. The inferior effect on growth parameters in the fertilisation groups with the lowest and highest wood ash ratios (1:1 and 4:1) indicates that the most appropriate mixture ratio for poplar fertilisation would be with one or two parts ash and one part digestate (2:1). The use of a suitable proportion is critical in natural and semi-natural ecosystems to prevent the threat of eutrophication. We also concluded that the origin of wood ash and the type of digestate did not have an impact on photosynthetic productivity. Therefore, a mixture made from various primary substrates of digestate and wood ash from various cogeneration plants can be used to create fertiliser, as long as it contains sufficient amounts of nutrients and comes from trusted sources to exclude unnecessary pollution by heavy metals. This study showed that wood chip cogeneration and biogas plant by-products—ash and digestate—can be used to achieve more productive tree plantations.

## Figures and Tables

**Table 1:** Fertilisation norms and amount of NPK nutrients incorporated into the soil depending wood ash and digestate ratios.

Primary Substrate of Digestate	Ash Origin	Ash and Digestate Mixture Ratio	Dry Mass of Digestate Proportion (kg ha^−1^)	Dry Mass of Ash Proportion (kg ha^−1^)	Dry Mass of Mixture Ratio (kg ha^−1^)	N (kg ha^−1^)	P_2_O_2_ (kg ha^−1^)	K_2_O (kg ha^−1^)
Control	Control	0:0	0	0	0	0	0	0
Pig manure digestate	a	1:1	3750	3750	7500	81.5	55.9	46.6
		2:1	3750	7500	11,250	88.4	89.6	80.4
		3:1	3750	11,250	15,000	74.1	114.0	126.6
		4:1	3750	15,000	18,750	70.5	146.4	168.2
	b	1:1	3750	3750	7500	84.6	60.8	29.0
		2:1	3750	7500	11,250	95.3	77.8	51.0
		3:1	3750	11,250	15,000	89.4	113.7	82.1
		4:1	3750	15,000	18,750	63.5	143.2	106.5
Cattle manure digestate	a	1:1	3750	3750	7500	NA	NA	NA
		2:1	3750	7500	11,250	NA	NA	NA
		3:1	3750	11,250	15,000	NA	NA	NA
		4:1	3750	15,000	18,750	NA	NA	NA
	b	1:1	3750	3750	7500	77.0	49.1	56.6
		2:1	3750	7500	11,250	60.7	80.0	90.8
		3:1	3750	11,250	15,000	61.9	111.1	136.4
		4:1	3750	15,000	18,750	60.3	145.6	169.6

Ash origin: a—LLC “Gren Latvia”, b—LLC “Dobeles Eko”.

## Data Availability

Data and code generated from this study are available upon request from the corresponding author.

## Appendix A

**Table A1:** Biogas digestate and wood ash mixture chemical composition, 29 April 2021.

Primary Substrate	Ash Origin	Mixture Ratios	Ph_CaCl2_	P Total (mg/g)	K (mg/g)	Ca (mg/g)	Mg (mg/g)	S (mg/g)	N (mg/g)
Pig manure digestate		0:1	7.0	7.3	19.2	28.8	13.2	22.1	470.1
Cattle manure digestate		0:1	7.6	5.4	13.9	18.3	5.7	18.35	456.0
	a	1:0	12.5	8.9	35.1	135.6	25.1	25.0	0.5
	b	1:0	12.3	8.0	114.2	290.2	78.7	52.0	0.3
Pig manure Digestate	a	1:1	9.3	8.1	38.7	154.1	37.9	43.2	11.3
		2:1	9.5	6.9	45.3	147.7	32.4	42.6	8.5
		3:1	9.8	7.6	54.7	189.2	44.9	51.8	6.0
		4:1	9.7	7.6	56.8	198.1	47.2	59.3	3.4
	b	1:1	9.6	7.5	62.1	187.2	40.3	27.9	10.9
		2:1	10.2	8.0	71.5	212.7	50.0	40.9	7.9
		3:1	11.5	7.6	84.4	258.6	65.5	44.6	4.9
		4:1	12.3	7.8	89.7	256.6	67.3	48.8	3.8
Cattle manure Digestate	a	1:1	NA	NA	NA	NA	NA	NA	NA
		2:1	NA	NA	NA	NA	NA	NA	NA
		3:1	NA	NA	NA	NA	NA	NA	NA
		4:1	NA	NA	NA	NA	NA	NA	NA
	b	1:1	9.6	6.6	75.5	191.3	40.2	32.3	10.3
		2:1	10.3	7.1	80.7	228.7	53.0	37.8	5.4
		3:1	11.8	7.4	90.9	236.3	57.5	43.7	4.1
		4:1	12.3	7.8	90.5	250.7	65.9	49.5	3.2
Forest soil	Control		4.3	0.3	4.4	9.64	0.9	3.4	92.5

K, C, Mg values are HCl extractable. Ash origin: a—LLC “Gren Latvia”, b—LLC “Dobeles Eko”.

## Appendix B

**Table A2:** Soil chemical composition at the end of the growth period, 26–30 August 2021.

Clone	Primary Substrate	Ash Origin	Mixture Ratios	pH_CaCl2_	P Total (mg/g)	K (mg/g)	Ca (mg/g)	Mg (mg/g)	N (mg/g)	C (mg/g)
AUCE	Pig manure digestate	a	1:1	3.6	0.2	5.1	18.6	6.8	3.5	76.7
			2:1	3.8	0.2	7.4	19.3	8.1	2.2	64.2
			3:1	4.8	0.3	5.6	27.8	10.4	2.4	67.0
			4:1	3.6	0.2	5.5	19.2	6.6	2.4	62.7
		b	1:1	3.6	0.4	5.8	22.2	7.5	3.5	80.6
			2:1	5.5	0.3	6.8	39.1	12.2	2.1	60.0
			3:1	4.2	0.2	6.5	22.7	8.3	2.3	69.8
			4:1	3.5	0.2	5.9	17.6	6.7	2.4	67.4
	Cattle manure digestate	a	1:1	3.5	0.3	4.7	22.5	7.2	2.9	76.0
			2:1	5.9	0.5	9.4	63.0	20.8	1.9	45.9
			3:1	3.5	0.1	3.7	14.0	4.8	2.0	53.6
			4:1	5.9	0.2	4.2	15.6	5.9	2.0	49.0
		b	1:1	3.5	0.1	3.9	11.6	4.5	2.4	57.2
			2:1	3.9	0.3	5.2	17.9	6.7	2.4	60.4
			3:1	3.9	0.2	3.7	17.0	6.0	2.5	70.0
			4:1	3.7	0.2	6.2	16.7	6.4	2.1	52.5
	Control			4.1	0.4	4.1	0.4	3.5	0.2	3.4
OP42	Pig manure digestate	a	1:1	4.1	0.2	4.9	23.6	8.8	3.3	87.6
			2:2	3.3	0.2	5.1	18.1	6.6	2.2	65.9
			3:1	3.6	0.2	4.6	15.5	5.8	2.0	55.7
			4:1	3.6	0.2	5.0	14.0	5.6	2.1	58.7
		b	1:1	3.6	0.3	4.7	18.6	6.5	3.5	77.3
			2:1	3.6	0.2	5.7	16.4	6.2	2.4	68.8
			3:1	6.7	0.6	11.0	10.4	29.4	2.9	84.4
			4:1	3.8	0.2	6.6	14.8	6.6	2.0	52.2
	Cattle manure digestate	a	1:1	4.0	0.3	4.6	25.0	9.6	2.3	61.3
			2:1	4.7	0.3	5.7	36.0	13.7	2.2	57.9
			3:1	3.5	0.2	4.3	16.7	7.4	2.8	76.4
			4:1	3.5	0.2	3.7	15.6	5.5	1.7	47.4
		b	1:1	3.6	0.1	4.1	15.5	5.9	3.2	93.1
			2:1	3.6	0.1	4.1	14.0	5.7	2.1	57.8
			3:1	3.7	0.5	11.1	67.9	20.1	3.0	81.8
			4:1	4.9	0.2	7.9	34.3	10.7	1.7	42.8
	Control			4.1	0.5	4.1	0.5	3.4	0.2	3.5

K, C, Mg values are HCl extractable. Ash origin: a—LLC “Gren Latvia”, b—LLC “Dobeles Eko”.

## Appendix C

**Table A3:** Chemical composition of leaves from 26–30 August 2021.

Clone	Primary Substrate	Ash Origin	Mixture Ratios	C (g/kg)	N (g/kg)	P Total (g/kg)
AUCE	Pig manure digestate	a	1:1	467.9	13.9	7.0
			2:1	470.5	14.2	5.0
			3:1	466.6	11.6	5.3
			4:1	467.4	12.6	4.5
		b	1:1	470.7	13.5	5.9
			2:1	465.5	17.7	5.9
			3:1	472.7	19.2	4.4
			4:1	486.0	16.5	3.3
	Cattle manure digestate	a	1:1	469.9	18.5	6.0
			2:1	472.2	14.7	5.1
			3:1	469.7	12.6	4.8
			4:1	474.5	12.3	3.7
		b	1:1	471.3	12.0	4.9
			2:1	468.2	14.3	5.3
			3:1	469.1	21.5	5.0
			4:1	472.6	13.2	3.8
OP42	Pig manure digestate	a	1:1	483.9	12.4	4.6
			2:2	483.9	13.5	4.3
			3:1	477.5	14.5	4.0
			4:1	487.6	10.1	2.2
		b	1:1	489.0	12.9	4.2
			2:1	480.2	11.2	3.2
			3:1	488.8	27.4	3.7
			4:1	479.7	10.8	2.2
	Cattle manure digestate	a	1:1	483.2	20.6	5.0
			2:1	485.6	15.1	4.3
			3:1	482.5	13.2	4.0
			4:1	487.4	12.9	3.4
		b	1:1	487.7	11.2	3.0
			2:1	484.8	11.5	3.5
			3:1	480.1	12.0	1.9
			4:1	489.4	24.3	2.7

Ash origin: a—LLC “Gren Latvia”, b—LLC “Dobeles Eko”.
